# From genes to drugs: *CYP2C19* and pharmacogenetics in clinical practice

**DOI:** 10.3389/fphar.2024.1326776

**Published:** 2024-02-14

**Authors:** Qamar Shubbar, Aminah Alchakee, Khaled Walid Issa, Abdul Jabbar Adi, Ali Ibrahim Shorbagi, Maha Saber-Ayad

**Affiliations:** ^1^ College of Medicine, University of Sharjah, Sharjah, United Arab Emirates; ^2^ Sharjah Institute for Medical Research, University of Sharjah, Sharjah, United Arab Emirates

**Keywords:** *CYP2C19*, pharmacogenetics, precision medicine, gentotype, pharmacoeconomic, CPIC, ethnic variation, gene polymorphism

## Abstract

The *CYP2C19* gene is frequently included in different pharmacogenomic panels tested in clinical practice, due to its involvement in the metabolism of a myriad of frequently prescribed medications. Accordingly, *CYP2C19* genotyping can promote precise therapeutic decisions and avoid the occurrence of significant drug-drug-gene interactions in the clinical setting. A comprehensive examination of the role of the *CYP2C19* gene in real-world medical settings is presented in this review. This review summarizes the most recent information on how genetic variants in *CYP2C19* affect drug metabolism and therapeutic outcomes. It goes into the wide range of CYP2C19 phenotypes, with different degrees of metabolizing activity, and their implications for customized medication response through a review of the literature. The review also analyzes the clinical significance of *CYP2C19* in several medical specialties, including cardiology, psychiatry, and gastro-enterology clinics, and illuminates how it affects pharmacological efficacy, safety, and adverse effects. Finally, *CYP2C19*-supported clinical decision-making is outlined, highlighting the possibility of improving therapeutic outcomes and achieving more affordable treatment options, a step towards optimizing healthcare provision through precision medicine.

## 1 Introduction

With the advancements in pharmacogenetics, *CYP2C19* has emerged as a gene for personalized drug prescriptions that serve many medical specialties, by considering the effect of genetic variants on the expected drug response (Naujokaitis et al., 2021). Therapeutic guidelines from the Clinical Pharmacogenetics Implementation Consortium (CPIC) are commonly used to recommend an appropriate treatment regimen based on the genotype test, specifically for patients in need of antiplatelet medication (Lee et al., 2022). Understanding the relevance of *CYP2C19* is essential for realizing the full promise of personalized medicine to improve medication use and transform contemporary healthcare (Zanger and Schwab, 2013). The insight gained from the diverse genetic alterations in *CYP2C19* and their consequences on enzyme action has ushered in a significant transformation in the approach to drug prescription and distribution. Healthcare professionals can maximize treatment efficacy while lowering the risk of adverse medication responses by implementing pharmacogenomic testing (Lee, 2013). This article will explore the practical applications of *CYP2C19* genotyping, particularly in handling cardiovascular (such as Clopidogrel), psychiatric (such as sertraline, fluoxetine, citalopram, escitalopram, and others), and gastrointestinal (such as Pantoprazole, Lansoprazole and Omeprazole (Esomeprazole)) conditions and drugs. The review aims to assess the role and impact of CYP2C19 status in current clinical settings. Furthermore, the challenges and benefits of integrating *CYP2C19* genotyping into healthcare strategies are discussed, considering elements such as cost-efficiency and prospective advances in personalized medicine. By meticulously analyzing both established literature and recent studies, this review seeks to contribute to the growing compendium of knowledge regarding the assimilation of *CYP2C19* genotyping into routine clinical practice.

## 2 Methodology of searching

Q.S. and Am.A. conducted an extensive search of PubMed and MEDLINE databases, covering literature from 2010 until October 2023. They independently screened titles, abstracts, and full texts to assess the eligibility of articles. The search methodology adhered to the Preferred Reporting Items for Systematic Reviews and Review Articles. The number of results was 44,613. However, after excluding review articles, animal studies and case reports, as well as duplications of data, they reached to the number of references used in this review article. The search strategy included the following relevant terms: “pharmacogenomics AND CYP2C19”, “clinical practice AND CYP2C19”, “ethnic variation AND CYP2C19”, “cardiology AND CYP2C19”, “psychiatry AND CYP2C19, “gastroenterology AND CYP2C19”. The inclusion criteria were English language, clinical trials, observational studies, pharmacokinetics studies, and epidemiological studies.

## 3 *CYP2C19* gene, and the role of CYP2C19 as a drug-metabolizing enzyme

The CYP450 superfamily is a large and diverse group of enzymes whose main function is to metabolize many drugs. CYP2C19 is a member of the CYP2C subfamily of cytochromes which are involved in the metabolism of a range of clinically important compounds, such as anticoagulants, proton pump inhibitors (PPIs), benzodiazepines, anticonvulsants, and tricyclic antidepressants (Saeed and Mayet, 2013). The drugs that are primarily metabolized by the CYP2C19 enzyme are listed in Table 1. The *CYP2C19* gene is located on chromosome 10q23.33 and to date, 39 alleles and 2000 SNPs have been identified (Shao et al., 2020). Among these variants, CYP2C19*2 and CYP2C19*3 are the most frequent and have received the greatest attention, as they identify poor metabolizers (Ionova et al., 2020) Understanding CYP2C19 as a metabolizing enzyme will enable healthcare professionals to make decisions regarding drug selection and dosing based on each individual’s genetic makeup to reach a safe and effective treatment (Pierre-François et al., 2022). Intriguingly, the *CYP2C19* gene is highly polymorphic, leading to changes in enzymatic activity, therapeutic responses, and/or adverse drug reactions. Within the CPIC guidelines, the system used to translate genotype to phenotype depends on the star (*) allele nomenclature (Botton et al., 2021). In particular, an individual is categorized as a normal [previously described as extensive metabolizer (EM)], intermediate metabolizer (IM), poor metabolizer (PM), rapid metabolizer (RM), or ultra-rapid metabolizer (UM) based on CYP2C19 metabolizing activity which is determined by their genetic profile. The most prevalent phenotype is the normal metabolizer with CYP2C19*1 genotype, in which individuals would be predicted to have full CYP2C19 functioning enzyme, allowing them to metabolize medicines efficiently. Poor metabolizers (PM) with CYP2C19*2 or CYP2C19*3 genotypes have restricted or absent CYP2C19 enzymatic activity, resulting in a delay in drug metabolism and probable drug toxicity, which can lead to unpleasant adverse effects. Intermediate metabolizers (IM) carry one loss-of-function allele such as the genotype *1/*2, allowing for intermediate drug metabolism. Ultra-rapid metabolizers (UM) with the CYP2C19*17 genotype, on the other hand, have overactive variations of the CYP2C19 gene, resulting in faster medication metabolism and clearance. Patients with ultra-rapid metabolizer phenotype may require higher doses of drugs to reach the intended therapeutic response (Rollinson et al., 2020).

**TABLE 1:** Drugs primarily metabolized by *CYP2C19*. Data obtained from PharmGKB.

Drugs primarily metabolized by *CYP2C19*
Gastrointestinal agents
Lansoprazole
Pantoprazole
Omeprazole (Esomeprazole)
Anti-infective agents
Voriconazole
Neurological and psychiatric agents
Benzodiazepine
Citalopram and Escitalopram
Clobazam
Clomipramine
Sertraline
Fluoxetine
Imipramine
Trimipramine
Doxepin
Cardiovascular and hematology agents
Clopidogrel
Pain and Anti-inflammatory agents
Cannabidiol

Drugs secondarily metabolized by *CYP2C19*
Psychiatric agents
Venlafaxine
Amitriptyline and Nortriptyline
Cardiovascular agents
Rosuvastatin
Neurological agents (with minor involvement of CYP2C19)
Clozapine
Paroxetine
Phenytoin
Venlafaxine
Analgesic and Anti-inflammatory agents
Methadone

## 4 *CYP2C19* testing

Pharmacogenomics (PGx) testing can involve a single gene or a panel of multiple genes. Evaluations of commercial PGx testing panels have revealed that gene composition as well as variant composition varies from test to test and frequently contains genes for which evidence is lacking to recommend prescription in various clinics, besides the fact that the *CYP2C19* gene is so large that, whole gene cannot be routinely performed. As a result, the number of genes on a testing panel is insufficient as a criterion for test selection. Even though the same genes appear on a testing panel, the number of sequence variants, or alleles, tested within those genes might vary significantly between tests. To achieve a high level of analytical validity (i.e., the capacity of a test to identify whether a certain genetic variation is present or missing), PGx testing should preferably be done in laboratories that have been certified and accredited under national regulations. According to the Clinical Laboratory Improvement Amendments (CLIA) and the College of American Pathologists (CAP), a validation process must be carried out to evaluate the accuracy, precision, reference interval, sensitivity, and specificity of a test (Black et al., 2020) When a person undergoes *CYP2C19* genetic testing, the analysis aims to identify and characterize specific genetic variations or alleles within the *CYP2C19* gene. The *CYP2C19* gene can have different forms, known as alleles, due to genetic variations among individuals. *CYP2C19* testing provides specific alleles. Different methods and approaches for *CYP2C19* testing are shown in Table 2.

**TABLE 2:** Comparing different methods of *CYP2C19* testing.

Method	Description	Time	Sample type	Cost	Available Kit/Assay (Company)
Genotyping (PCR-based test), some will require subsequent DNA/Sanger sequencing	Identifies specific *CYP2C19* alleles using polymerase chain reaction	1–3 days	Blood	$357 to $1,230	TaqMan™ SNP Genotyping Assay, human (Thermo Fisher Scientific)
				-	xTAG^®^ *CYP2C19* Kit v3 (Luminex)
				-	gb PHARM *CYP2C19* (Generi Biotech)
				-	*CYP2C19* Genotyping Diagnostic Kit (PCR-Fluorescence Probing) (DaAnGene)
				-	Mutector™ *CYP2C19* Genotyping kit (TrimGen)
DNA sequencing	Comprehensive analysis of the entire *CYP2C19* gene	1–2 weeks		High	-
NGS (Next-generation sequencing)	High-throughput DNA sequencing to analyze multiple genes	1–2 weeks		High	-
Point-of-Care Testing	Rapid on-site testing for immediate results in emergency settings	Few hours		Moderate	INFINITI *CYP2C19* Assay (AutoGenomics)

## 5 *CYP2C19* genotype-guided therapy (individualized prescription)

Genotype-guided treatment, also known as tailored prescription, is an advanced strategy in personalized medicine that optimizes drug selection and administration based on a patient’s exact genetic composition. By evaluating genetic variations of the *CYP2C19* gene, healthcare practitioners can determine an individual’s drug metabolism phenotype and classify them as normal metabolizer (NM), poor metabolizer (PM), intermediate metabolizer (IM), rapid metabolizer (RM) or ultra-rapid metabolizer (UM) (El Rouby et al., 2018)Clinicians may adjust medicine prescriptions based on a patient’s specific metabolic profile, ensuring that patients receive the most effective treatment, safely. Genotype-guided treatment is very useful when prescribing drugs with proven *CYP2C19* involvement, such as clopidogrel for cardiovascular illnesses or PPIs for digestive disorders. *CYP2C19* genotype-guided drug prescribing has been proven in several trials to increase pharmacological effectiveness and safety. For example, CYP2C19 PMs have been observed to have a greater risk of cardiovascular events in patients receiving antiplatelet treatment with clopidogrel following stent installation. Switching PMs to alternative antiplatelet medications, such as ticagrelor or prasugrel, based on their genotype, has been associated with better clinical outcomes (Castrichini et al., 2023). A summary of studies on *CYP2C19* Genotype-Guided therapy in cardiovascular practice is shown in Table 3. *CYP2C19* genotype-guided medication has the potential to increase treatment efficacy, minimize adverse drug responses, and improve overall patient outcomes by incorporating genetic information into the treatment decision-making process, ushering in a new age of personalized medicine. Other aspects, such as drug interactions, co-existing illnesses, and lifestyle issues, must also be considered to achieve thorough and holistic patient treatment.

**TABLE 3:** *CYP2C19* genotype-guided therapy in cardiology practice.

Study/Clinical trial	Number of Participants	Condition	Findings	Therapy received	References
Diverse Clinical Settings	3,342	Percutaneous Coronary	CYP2C19 LOF carriers treated with alternative therapy had lower atherothrombotic risk compared to clopidogrel and similar risk in those with non-LOF allele treated either with clopidogrel or alternative therapy	Alternative therapy with prasugrel or ticagrelor compared to Clopidogrel	Beitelshees et al. (2022)
		Intervention			
Single-center observational cohort study (China)	1,361	Percutaneous coronary intervention	The MACCE rate was higher in the LOF-clopidogrel group compared with the LOF-ticagrelor group (p = 0.029). No significant difference in the incidence of MACCE, in non-LOF-clopidogrel compared to LOF-ticagrelor group (p = 0.272)	Ticagrelor compared to Clopidogrel	Zhang et al. (2021)
TAILOR-PCI Randomized Clinical Trial (US, Canada, South Korea, and Mexico)	5,302	Percutaneous coronary intervention (for acute coronary syndromes (ACS) or stable coronary artery disease (CAD))	Genotype-guided therapy of P2Y12 inhibitor, compared with conventional clopidogrel therapy, showed no statistically significant difference	Ticagrelor and noncarriers clopidogrel compared to Clopidogrel	Pereira et al. (2020)
Real-world cohort (University of North Carolina-Chapel Hill)	1,063	Percutaneous Coronary Intervention	Compared to alternative therapy, CYP2C19 LOF allele carriers receiving clopidogrel showed a significantly higher risk of MACCE or bleeding over 30 days	Genotype-guided prescribing, ticagrelor or prasugrel	Williams et al. (2019)
Single-center, non-randomized, retrospective cohort study (China)	1,134	Off-pump coronary artery bypass grafting (OPCAB)	individual DAPT (CYP2C19 genotype with platelet aggregation test) was associated with lower risk of MACE and a similar risk of major bleeding compared to traditional DAPT (Aspirin with Clopidogrel)	Dual antiplatelet therapy (DAPT)	Yao et al. (2022)
Prospective, open-label RCT	650	Ischemic stroke or transient ischemic attack (TIA)	Clopidogrel guided therapy can significantly improve the overall clinical benefit of ischemic stroke or TIA patients without increasing the risk of bleeding	The pharmacogenetic group received aspirin combined with clopidogrel/ticagrelor based on clinical characteristics. While the standard group received aspirin combined with clopidogrel	Zhang et al. (2023)

## 6 *CYP2C19* in clinical practice

### 6.1 *CYP2C19* in cardiology

i. Significant recommendations in clinical practice according to the gene variant:

*CYP2C19* gene variants play an important role in cardiology, primarily considering the metabolism of commonly used medications. These gene variants can significantly influence how patients metabolize drugs, especially, clopidogrel, a widely used antiplatelet drug (Pereira et al., 2019). Poor metabolizers may exhibit reduced conversion of clopidogrel to its active form, thus compromising its effectiveness in preventing cardiovascular events. In consideration of this, cardiology practice recommendations emphasize the importance of genotyping *CYP2C19* variants to identify patients at higher risk and personalize their medications accordingly (Turner and Pirmohamed, 2014). On the other hand, concomitant use of clopidogrel and PPIs may reduce the activation and it will affect the efficacy of clopidogrel, hence the need for clinicians to consider PPIs with minimal *CYP2C19* dependent (Kenngott et al., 2010). Table 4 summarizes *CYP2C19* gene variation based on the following recommendations that are relevant in the cardiology clinic.

ii. Recommended panels:

**TABLE 4:** Dosing recommendations for clopidogrel based on *CYP2C19* phenotype. Data with strong evidence was retrieved from CPIC guidelines.

Phenotype	Drug	Haplotype	Result	Therapeutic recommendation	Classification	References
Ultrarapid metabolizers	Clopidogrel	*17/*17	Due to increased metabolism, it will result in a lower antiplatelet reactivity, and no association with bleeding risk	If using Clopidogrel, use the standard dose of 75 mg	Strong	Lee et al. (2022)
Rapid metabolizer		*1/*17	Normal or increased metabolism of the drug, and no association of bleeding risk			
Intermediate metabolizer		*1/*9, *9/*17, *9/*9	Due to reduced metabolism, lower platelet reactivity will result, increasing the risk of cardiovascular events	Avoid the standard dose of Clopidogrel and use Prasugrel or Ticagrelor at standard dose if no contraindication	Moderate	
Poor metabolizer		*2/*9, *3/*9	Significantly reduced clopidogrel metabolism, increased risk of cardiovascular risk	Avoid Clopidogrel and if no contraindication, use Prasugrel or Ticagrelor		

When considering whether to include *CYP2C19* genotyping with other genes in the cardiology clinic, it is vital to concentrate on genes that can alter how drugs are metabolized, especially for medications that are often prescribed. Together with *CYP2C19*, several genes may be taken into consideration for panels, such as *CYP2C9*, another essential gene in drug metabolism that is particularly important for drugs like warfarin and non-vitamin K oral anticoagulants (NOACs). *VKORC1* is another crucial gene, as it influences the dose needed to achieve the desired anticoagulation effect of warfarin. Combining *CYP2C19*, *CYP2C9*, and *VKORC1* genotyping helps researchers to have a full picture of how patients metabolize these drugs (De Lara et al., 2022). The *ADRB1* gene, which encodes for the beta-1 adrenergic receptor, is one of the genes that have an impact on the response of beta blockers which are commonly used in the treatment of heart failure and hypertension. Considering patients’ *ADRB1* genotype will provide clinicians with guidance for the selection and dose of these medications (Howaidi and Lababidi, 2022). The *ADRA2A* gene which encodes for alpha-2A receptor, has a vital role in the regulation of the sympathetic nervous system. Variation in the *ADRA2A* gene can also influence the patient’s response to beta-blocker drugs. The precision of cardiovascular therapy could be improved by including these genes in a curated panel as it would enable a more individualized approach that considers a person’s genetic variables, lowering the chance of adverse reactions and ensuring that patients receive the best possible care.

iii. Adverse effects:

In a study on 168 patients with coronary heart disease who received clopidogrel/dual antiplatelet therapy (DAPT) after percutaneous coronary intervention (PCI), the incidence of cardiovascular adverse events was recorded by the high-on treatment-platelet reactivity (HPR) at 1-year follow-up visits. HPR was measured using thrombo-elastography which is a test used to assess the efficiency of blood coagulation. Moreover, PCR was done at the beginning of the study to determine *CYP2C19* and *ABCB1* 3435^−ΔΔCT^ gene polymorphisms. The study concluded that the non-functional CYP2C19*3 variant was associated with a higher incidence of HPR which was correlated with a higher incidence of cardiovascular adverse events. On the other hand, the non-functional allele CYP2C19*2 and *ABCB1* 3435^−ΔΔCT^ were not significantly associated with HPR or cardiovascular events. This suggests that *CYP2C19* and *ABCB1* 3435^−ΔΔCT^ genotyping before initiation of clopidogrel therapy can be a significant predictive factor for treatment failure and the development of adverse effects (Mega et al., 2010). DAPT with aspirin and a P2Y12 inhibitor (clopidogrel, prasugrel, or ticagrelor) is known to be the standard care therapy for patients following PCI. The CYP2C19 enzyme is responsible for the activation of the prodrug “clopidogrel” into its active metabolite to carry out its antiplatelet activity. Patients who carry the non-functioning allele of *CYP2C19* and receive clopidogrel as anti-platelet therapy are at a higher risk of treatment failure and development of major adverse cardiovascular and cerebrovascular events. Prasugrel and ticagrelor have not been linked with *CYP2C19* activation like clopidogrel, which makes them better options for patients with non-functioning *CYP2C19* alleles. Therefore, *CYP2C19* genotype-guided anti-platelet therapy is believed to be beneficial for the prevention of major adverse cardiovascular and cerebrovascular events, which is further reinforced by multiple data emerging from cardiovascular and neurology clinical studies (Sanderson et al., 2005). Multi-factorial drug-gene interaction is the umbrella term used to describe the cumulative effects of both drug-drug interactions and drug-gene interactions. This phenomenon can be applied to patients who inherited the *CYP2C19* loss-of-function allele and are receiving clopidogrel with concomitant PPI administration. In a systematic review and meta-analysis, five studies were included, comprising 8,802 patients of coronary heart disease or stroke. 3,767 were prescribed clopidogrel alone, 1,931 were concomitantly taking clopidogrel and PPIs, 2,146 were carrying *CYP2C19* loss-of-function alleles and 958 were taking both clopidogrel and PPIs while also carrying *CYP2C19* loss-of-function alleles. Patients with coronary heart disease or stroke who are receiving clopidogrel and concomitant proton pump inhibitor (PPI) therapy while inheriting loss-of-function alleles (CYP2C19*2 or CYP2C19*3) had a 63% higher risk of developing major cardiovascular adverse events (Biswas et al., 2021). In another systematic review and meta-analysis, 12.2% carried the CYP2C9*2 variant and 7.9% carried the CYP2C9*3 variant. Previous reports showed a 17% reduction in the original warfarin dose for CYP2C9*2 carriers and 37% for CYP2C9*3 carriers with a relative bleeding risk of 1.91 and 1.77, respectively. The study concluded that patients who carry CYP2C9*2 and CYP2C9*3 have a lower mean daily warfarin dose and a higher bleeding risk, which speculates that genotype-guided warfarin therapy could markedly alter the management of patients being started on warfarin (Barbarino et al., 2018). However, it showed that there is a marked difference in the enzymatic activity between CYP2C9*2 and CYP2C9*3 carriers. Poor metabolizer patients on Clopidogrel carrying CYP2C19 *3/*9 genotype may experience diminished antiplatelet effects, potentially increasing the risk of cardiovascular events, thus, they may use alternative drugs such as Prasugrel or Ticagrelor. The diplotype is more commonly seen in African American/Afro-Caribbean and Sub-Saharan African populations as the frequency is 0.01% according to CPIC, while in Latino population its frequency is 0.0001%. Such meta-analyses signify the critical role of *CYP2C19* as a key pharmacogene in cardiology practice.

iv. Drug-drug interactions:

Co-occurrence of drug-drug interaction (DDI) with drug-gene interaction (DGI) might alter drug biotransformation pathways and produce drug-drug-gene-interaction (DDGI). PPIs are commonly used in the treatment of gastric disorders. Omeprazole is among the PPIs which poses the highest propensity to interact with other drugs compared to other PPIs like pantoprazole, rabeprazole, and lansoprazole. This is explained by its high affinity for CYP2C19 and moderate affinity for CYP3A4. Studies published since 2006 have shown clinically significant interaction between the antiplatelet medication, clopidogrel, and omeprazole which is mediated by CYP2C19 (Barbarino et al., 2018). Patients receiving DAPT often receive PPI therapy to reduce the risk of bleeding. Clopidogrel, the antiplatelet agent most commonly used in DAPT poses a challenge when concomitant PPI therapy is given due to their conflicting pharmacokinetic interaction *via* CYP2C19. Even though concomitant use of PPI and DAPT has been shown to decrease active metabolites of clopidogrel and ex vivo-measured platelet inhibition, there is still a conflict about whether this interaction has a significant effect on clinical outcomes (Saven et al., 2022).

### 6.2 *CYP2C19* in psychiatry

i. Significant recommendations in clinical practice according to the gene variant:

The CYP2C19 enzyme plays a crucial role in the metabolism of many antidepressants, including selective serotonin reuptake inhibitors (SSRIs) such as sertraline, fluoxetine, citalopram, escitalopram, and others. In addition, multiple conventional tricyclic antidepressants such as imipramine, amitriptyline, trimipramine and clomipramine, are known CYP2C19 substrates (Alchakee et al., 2022). Genetic variations of *CYP2C19* significantly impact the efficacy and safety of antidepressant medications, thus clinically influencing depression management. Table 5 summarizes dosing recommendations of antidepressants classified as level 1A evidence. Level 1A evidence indicates a specific gene-variant prescribing advice is provided in current clinical guidelines or FDA-approved drug label annotations (Hicks et al., 2015). Based on the *CYP2C19* genotype, the CPIC published gene-based therapy recommendations for the SSRIs citalopram and escitalopram. For CYP2C19 ultrarapid and poor metabolizers, it is recommended to use an alternative antidepressant that is not primarily metabolized by CYP2C19 or to adjust the dose according to metabolizer status. Furthermore, people with a *CYP2C19* *17/*17 genotype have significantly lower citalopram or escitalopram plasma concentrations at steady state when compared to normal metabolizers, thus it is recommended to titer citalopram to a higher target dose (compared to normal metabolizers) or to initiate an alternative SSRI, such as fluoxetine, fluvoxamine, and paroxetine, which are strongly metabolized by CYP2D6 only (Wong et al., 2023). The choice of an alternative antidepressant medication should be individualized based on the patient’s specific needs and medical history.

ii. Recommended panels:

**TABLE 5:** Dosing recommendations for antidepressants based on *CYP2C19* phenotype. Data with strong evidence were retrieved from PharmGKB and CPIC guidelines.

Drug name	Genotype	Phenotype	Dose recommendation	Alternative Antidepressant
SSRI				
Es-/citalopram	*17/*17	Ultra-rapid	consider titrating to a higher maintenance dose	Fluoxetine, Fluvoxamine, Paroxetine. (Nikolac Perkovic et al., 2020)
	*2/*9, *3/*9	Likely Poor Metabolizer	Per the FDA warning, citalopram 20 mg/day is the maximum recommended dose in CYP2C19 poor metabolizers due to the risk of QT prolongation	
	*2/*2, *2/*3, *3/*3	Poor metabolizers	citalopram dose should be limited to 20 mg/day in patients with hepatic impairment, those taking a CYP2C19 inhibitor, and patients greater than 60 years of age	
Sertraline	*17/*17	Ultra-rapid	Initiate therapy with the recommended starting dose	
	*1/*17	Rapid Metabolizer	Initiate therapy with the recommended starting dose	
Tricyclic Antidepressants (TCAs)				
Amitriptyline	*1/*2, *1/*3, *2/*17	Intermediate metabolizer	Initiate therapy with the recommended starting dose	Bupropion, Fluvoxamine, Mirtazapine or Paroxetine (Kee et al., 2023)
Clomipramine	*1/*1	Normal metabolizer	Initiate therapy with the recommended starting dose	
Imipramine				
Trimipramine				

The cytochrome P450 isoenzymes, mainly CYP2D6, CYP2C9, and CYP2C19 are responsible for the metabolism of the majority of psychotropic medications, including antipsychotics, antidepressants, and mood stabilizers. The highly polymorphic CYP2C19 enzyme plays a crucial role in the metabolism of many antidepressants, including SSRIs such as, sertraline, fluoxetine and (es)citalopram. Psychiatric gene sequencing panels vary depending on the specific focus of a clinic, patient population, psychiatric disorders, or medications of interest (Thiele et al., 2022). CYP2C19 demethylates several tricyclic antidepressants including, clomipramine, amitriptyline, trimipramine and imipramine to pharmacologically active metabolites. These compounds and their metabolites along with nortriptyline and desipramine, are hydroxylated by CYP2C19 enzyme to fewer active metabolites (Hicks et al., 2013). Therefore, combining *CYP2D6* and *CYP2C19* genomic variants in a single panel can provide a more comprehensive understanding of an individual’s drug metabolism profile that affects drug efficacy and safety (Matthaei et al., 2021). Interestingly, in psychiatry, *ADRB1* polymorphisms, along with other genes, increase the risk of developing Alzheimer’s disease and sleep disturbances caused by altered cell responsiveness to adrenergic stimulation (Bullido et al., 2004). Genetic variations of the catechol-O-methyltransferase (*COMT*) gene, which encodes for an enzyme involved in the metabolism of dopamine and norepinephrine, have been associated with altered response to antipsychotic medications (Nikolac Perkovic et al., 2020). Notably, some studies in cardiology have explored a relationship between *COMT* variants and hypertension; however, data on this is scarce, and further investigations are required (Xu et al., 2017). In general, combining *CYP2C19* with other actionable pharmacogenes in a genotyping test can provide valuable insights into personalized medicine.

iii. Adverse effects:

The discontinuation of antidepressant treatment is a common behavior in people with depression, mainly due to adverse drug reactions. Nearly 50% of undesirable drug reactions can be attributed to the differences in drug metabolism between individuals (Solomon et al., 2019; Kee et al., 2023). A clinical study was conducted to explore the association of *CYP2C19* actionable variants translated into phenotypes with suicidal behavior in patients with depression who were using citalopram. The rate of suicide was 2-fold higher in individuals classified as CYP2C19 poor metabolizers compared to those classified as CYP2C19 normal metabolizers (Aldrich et al., 2019; Joas et al., 2023). The association of CYP2C19 metabolism status and side effects including hyperactivity, weight gain, gastrointestinal symptoms and insomnia was also investigated in pediatric patients prescribed escitalopram for anxiety or depressive disorders. The CYP2C19 poor metabolizers experienced more unwanted effects compared to faster metabolizers. In particular, CYP2C19 PMs had more rapid weight gain and hyperactivity (Ramsey, 2018). A recent Australian study consisting of 9,500 participants revealed that escitalopram is more tolerable by rapid CYP2C19 metabolizers while sertraline is more tolerable by poor CYP2C19 metabolizers, compared to normal metabolizers (Campos et al., 2022). On the other hand, a Swedish genetic study has revealed that the incidence of treatment-emergent mania was increased in patients with slower CYP2C19 metabolism status who were using amitriptyline or sertraline to treat bipolar depression with a hazard ratio (1.3, 1.46), respectively (Rahikainen et al., 2019). The Pre-emptive Pharmacogenomic Testing for Preventing Adverse Drug Reactions (PREPARE) study is the first large-scale and randomized clinical trial conducted in Europe to investigate the impact of applying pharmacogenomic test on the incidence of adverse drug reactions. The PREPARE study covered 39 different medications to treat multiple diseases. Notably, preemptively tested participants with actionable variants experienced a remarkable 30% reduction in the incidence rate of clinically relevant adverse drug reactions associated with drug-genotype interactions (Swen et al., 2023). Therefore, determining the patient’s CYP2C19 metabolizing status based on his genetic profile might enhance the safety of using antidepressant medications (Joas et al., 2023).

iv. Drug-drug interactions:

As previously mentioned, significantly altered rates of metabolism may occur due to DDGI. Escitalopram is mainly metabolized by the CYP2C19 and CYP3A4 enzymes and to a lesser extent by the CYP2D6 enzyme. Blood concentration of escitalopram is significantly influenced by the concomitant administration of *CYP2C19*, *CYP3A4*, and *CYP2D6* modulator drugs (Rochat et al., 1997). The Combination of *CYP3A5* and *CYP2C19* genetic polymorphisms mediates several DDIs and DGIs. For instance, the co-presence of CYP3A4 EM and CYP2C19 IM/PM increases the risk of (es)citalopram toxicity and hence the urge for dose reduction or drug switching (Bahar et al., 2020). Unfortunately, the recent dosage recommendation for escitalopram is based on DGIs and DDIs separately and a knowledge gap remains regarding In a recent clinical case report, a patient complained of inadequate depression control despite several attempts with multiple antidepressants, including escitalopram, venlafaxine, and bupropion. The patient was phenotypically a *CYP2C19* IM and a *CYP2D6* PM (due to phenoconversion), and genetic variants in *CYP2D6* and *CYP2C19* increased their venlafaxine plasma concentration. In addition, the metabolism of other concomitant medications was impacted by the strong *CYP2C19* inhibitor, bupropion, which contributed to the treatment failure. Cannabidiol (CBD) and PPIs are clinically known as *CYP2C19* inhibitors and hence cause *CYP2C19* phenoconversion (Von Moltke et al., 2001; Bousman et al., 2023). In particular, the concomitant use of CYP2C19 inhibitors and psychiatric medications may commonly lead to phenotype conversion from nonpoor metabolizer phenotype to poor metabolizer phenotype (Klieber et al., 2015). In a recently published case report, a patient with intermediate CYP2C19 phenotype who was on sertraline for 20 years developed cognitive dysfunction and hyponatremia due to an increase in sertraline plasma concertation after addition of CBD to their treatment regimen (Nanan et al., 2022). In another clinical case report, a patient complained of inadequate depression control despite several attempts with multiple antidepressants, including escitalopram, venlafaxine, and bupropion. The patient was phenotypically a CYP2C19 IM and a CYP2D6 PM, and genetic variants in *CYP2D6* and *CYP2C19* increased his venlafaxine plasma concentration. In addition, the metabolism of other concomitant medications was impacted by the strong CYP2C19 inhibitor, bupropion, which contributed to the treatment failure (Nanan et al., 2022).

### 6.3 *CYP2C19* in gastroenterology

i. Significant recommendations in clinical practice according to the gene variant:

Based on the previously described *CYP2C19* gene variation, recommendations relevant to Gastroenterology are summarized in Table 6. Individual patient variables, pharmacological interactions, and the exact clinical circumstance should all be considered when deciding on the best treatment approach. PPI dosing recommendations based on CYP2C19 phenotype reflect a prototypical integration of pharmacogenomics into gastroenterology as shown in Table 7. CYP2C19 is a key enzyme in PPI metabolism, *CYP2C19* genetic variations have important implications for the therapeutic response and safety of PPI regimens. Substantial data compiled by impactful sources such as PharmGKB (Pharmacogenomics Knowledgebase) and the stringent clinical directions provided by the CPIC guidelines contribute to the cogency of these suggestions.

ii. Recommended panels:

**TABLE 6:** Recommendations based on *CYP2C19* gene variants in the Gastroenterology clinic.

Phenotype	Drug	Reason of using the drug	Haplotype	Result	Alternative	References
Poor metabolizers	Omeprazole/Pantoprazole	Different PPI’s indications	*2/*2, *2/*3, *3/*3	Due to decreased metabolism, the efficacy will be reduced	Rabeprazole, which is less dependent PPI on *CYP2C19*	Lima et al. (2021)
	Clopidogrel	Cardiovascular patients with gastrointestinal issues		Reduced effectiveness of Clopidogrel and increase in cardiovascular adverse effects	Ticagrelor or Prasugrel, are used as they are not influenced by *CYP2C19*	Lee et al. (2022)
	Escitalopram	Manage some gastroenterological conditions associated with anxiety or depression		Higher drug levels, so increased risk of side effects	Dosing adjustments or consider alternative antidepressant medications	Bousman et al. (2023)

**TABLE 7:** Dosing recommendations for Proton Pump Inhibitors based on *CYP2C19* phenotype. Data with strong evidence were retrieved from PharmGKB and CPIC guidelines.

Drug name	Diplotype	phenotype	Dose recommendation
Omeprazole, Lansoprazole, Pantoprazole, and Dexlansoprazole	*17/*17	Ultra-rapid Metabolizer	Increase starting daily dose by 100%
	*1/*17	Rapid Metabolizer	Initiate standard starting daily dose. Consider increasing the dose by 50%–100% for the treatment of *Helicobacter pylori* infection and erosive esophagitis
	*1/*1	Normal metabolizer	Initiate standard starting daily dose
	*1/*2, *1/*3, *2/*17, *3/*17	Intermediate Metabolizer	Initiate standard starting daily dose. For chronic therapy (>12 weeks) and efficacy achieved, consider 50% reduction in daily dose
	*2/*2, *3/*3, *2/*3	Poor metabolizer	

Genetic testing panels at a gastrointestinal clinic can offer useful information about patients' genetic tendencies and probable drug reactions. Genetic testing can be used to personalize therapy regimens, forecast illness risk, and spot potential negative effects. Although several genetic testing panels could be taken into consideration, advice on the use of particular panels may vary depending on the clinic’s specialty and resources. The Inflammatory Bowel Disease (IBD) Panel, which includes *NOD2*, *IL23R*, and *ATG16L1* genes along with *CYP2C19*, which are associated with IBD severity and susceptibility can aid in defining genetic risk and tailoring treatment for patients suffering from Crohn’s disease and ulcerative colitis (Slavin et al., 2019). The Liver Disease and Drug Metabolism Panel which includes *CYP2C19*, *UGT1A1*, *HFE*, and other genes, covers genetic variables impacting liver disorders and drug metabolism, which are relevant in gastrointestinal (Liu et al., 2022) and could be very relevant to clinical practice due to the liver’s crucial function in digestion and drug processing. A collaborative approach could provide an understanding of the intricate genetic framework that underpins gastrointestinal wellbeing, enabling healthcare professionals to define patient-focused alignment of diagnostic and therapeutic strategies.

iii. Adverse effects:

Adverse effects pose a significant concern in the Gastroenterology clinic, and understanding both genophenotypic factors and pharmacogenomics can provide insights into the origins of adverse drug reactions (ADRs). Genophenotypic factors encompass genetic and phenotypic variations that significantly affect an individual’s susceptibility to ADRs. Meanwhile, pharmacogenomics investigates how genetic variants affect medication metabolism, effectiveness, and safety (Chevalier et al., 2023). Adverse consequences of *CYP2C19* genetic variants can emerge as impaired drug metabolism and reactions to drugs routinely used in gastrointestinal diseases. The most prevalent side effect related to *CYP2C19* genetic variants is an altered response to PPIs in poor metabolizers carrying *CYP2C19* loss-of-function variants which leads to reduced efficacy of some PPIs like omeprazole and lansoprazole), due to impaired conversion to their active forms. This would result in diminished acid suppression, which will potentially impact symptom relief of gastrointestinal disorders such as peptic ulcer disease, and gastroesophageal reflux disease (GERD) (Paré et al., 2010). For patients with gastrointestinal and cardiac conditions, CYP2C19 poor metabolizers may have a reduced ability to convert clopidogrel to its active form, thus resulting in decreased antiplatelet activity, and leading to increased adverse cardiovascular events (Chen et al., 2012). Patients with known *CYP2C19* genetic variants should be actively examined by gastroenterologists for the risk of these unfavorable outcomes. To minimize these side effects and improve treatment results, genetic testing can disclose important information about a patient’s metabolic profile, which can then be used to guide medication selection and dosing modifications.

## 7 Pharmacoeconomic (health burden) and *CYP2C19*

Pharmacoeconomic studies assessing the effect of *CYP2C19* genotype-guided treatment have garnered attention in recent years, the purpose of which is to analyze the economic consequences of using pharmacogenetic tests to guide pharmacological therapy selections based on *CYP2C19* genetic variations. Pharmacoeconomic studies evaluate the economic effect of various treatment regimens by considering both direct hospital expenses and larger social costs associated with illness management (Sorich et al., 2013). In a meta-analysis of pharmacoeconomic research on *CYP2C19* genotype-guided antiplatelet medication in patients with acute coronary syndrome, researchers discovered that genotyping individuals and tailoring antiplatelet medication based on *CYP2C19* variations resulted in significant reductions in severe adverse cardiovascular events and total healthcare expenditures (Fu et al., 2019). Lee et al. assessed the cost-effectiveness of *CYP2C19* genotype-guided antiplatelet treatment in patients undergoing PCI. The study found that genotype-guided medicine was a more cost-effective choice than the standard treatment, particularly in those at high risk for adverse cardiovascular events. The study highlighted the significance of incorporating pharmacogenetic testing into standard clinical practice to improve treatment results and resource use (Lee et al., 2011). Another research on the cost-effectiveness of *CYP2C19* genotype-guided antiplatelet treatment in Korean patients having PCI for acute coronary syndrome found that adding genotyping into clinical decision-making was a cost-effective strategy that resulted in improved clinical outcomes and decreased healthcare costs when compared to standard therapy. The authors emphasized the potential for significant economic gains from genotype-guided treatment (Al-Rubaish et al., 2020). Overall, data suggests that *CYP2C19* genotype-guided therapy has the potential to improve patient outcomes and reduce healthcare costs in a range of clinical contexts, notably antiplatelet treatment for cardiovascular cases. Healthcare professionals can personalize medication therapies to optimize therapeutic advantages while avoiding adverse drug responses and treatment inefficiencies by identifying patients with distinct *CYP2C19* variations. Despite the positive data, broad implementation of pharmacogenetic testing in ordinary clinical practice remains a challenge, and further research is needed to overcome adoption obstacles and enable fair access to genotype-guided medication. As the field of pharmaco-economics gains more attention, more research and real-world data will be needed to drive policy and procedure that is grounded in evidence in personalized medicine. In another research study, the authors propose that PGx-guided clopidogrel therapy is an affordable choice for ACS patients receiving care in Spain (Koufaki et al., 2023).

## 8 Ethnic variation

In the framework of *CYP2C19* genetics and its impact on drug metabolism, ethnic diversity is crucial to explore. Normal metabolizers (NMs) may be more prevalent in some cultures, whilst poor metabolizers (PMs) may be more prevalent in others. Within certain ethnic groups, these variances may affect pharmaceutical reactions and efficacy. It is essential to understand ethnic diversity in *CYP2C19* genotypes to tailor pharmacological regimens and improve treatment results while taking into consideration genetic propensity and sensitivity to adverse drug responses (Nguyen et al., 2022). In a study conducted to evaluate the disparity between individuals with different racial backgrounds, when it comes to *CYP2C19* genotype-guided P2Y12 antiplatelet therapy, patients from 9 sites that performed genotyping for *CYP2C19* following percutaneous coronary intervention was recruited. A total of 3,342 participants were included, out of which 2,448 (73%) were European people and 659 (20%) were African people. The main aim was to compare the rate of prescribing P2Y12 inhibitors between European and African people races following *CYP2C19* genotyping to guide antiplatelet therapy selection after PCI. Patients who carried the non-functioning *CYP2C19* allele were prescribed alternative P2Y12 inhibitors (Prasugrel and Ticagrelor) instead of clopidogrel since clopidogrel’s effectiveness would be decreased. Choosing between clopidogrel and alternative therapy based on the genotype was the primary outcome. African people had a significantly higher prevalence of carrying the non-functioning allele compared to European people. There was no statistically significant association between race (European and African people with non-functioning alleles) and the prescription of alternative antiplatelet therapy at discharge following PCI and 12 months after the last follow-up visit. According to this study, there is an absence of racial disparity in genotype-guided antiplatelet prescribing among patients receiving *CYP2C19* testing (Cavallari et al., 2023). The clinical outcomes of the coadministration of clopidogrel and omeprazole have not been adequately studied in the Asian population. It is believed that concomitant administration of omeprazole decreases the efficacy of clopidogrel due to its inhibition of the CYP450 *CYP2C19* variant, which is responsible for the activation of clopidogrel. According to several studies, this interaction has not shown an increase in mortality or incidence of myocardial infarction in Caucasians. Data are scarce regarding this combination of drugs in the Asian population, which is believed to have a high prevalence of the non-functioning allele of *CYP2C19*. In a retrospective study that utilized the medical records and prescriptions of more than12,000 Asian patients receiving clopidogrel. The study findings revealed that coadministration of clopidogrel and omeprazole had a significant positive association with the incidence of MI, but the association with mortality, cerebrovascular accidents, and coronary interventions deemed to be statistically insignificant. Additionally, there was ethnic variability, with an increased incidence of MI in the Malay and Chinese populations compared to the Indian population (Muthiah et al., 2021). East-Asian populations commonly exhibit a higher prevalence of the CYP2C19*17 allele, a genetic variant associated with increased enzymatic activity, leading to ultra-rapid drug metabolism. However, this genetic trait has implications for the use of proton pump inhibitors (PPIs). The increased enzymatic activity associated with the CYP2C1917 allele may result in faster metabolism of PPIs, potentially leading to reduced drug efficacy (Zhang, 2021). In Oceanian populations, there is a notable increase in the allele frequency of CYP2C19*2 and CYP2C19*3 genetic variants, contributing to a higher prevalence of individuals classified as poor metabolizers of clopidogrel. However, the drug’s activation is heavily dependent on the enzymatic activity of CYP2C19. The *2 and *3 alleles are associated with reduced function of the CYP2C19 enzyme, leading to impaired conversion of clopidogrel into its active form (Helsby, 2016).

## 9 Conclusion

This review article emphasizes the importance of *CYP2C19* in clinical practice across a range of various disciplines. *CYP2C19* genotypes should be considered when prescribing drugs in Cardiology and Gastroenterology clinics, such as antiplatelet medicines and PPIs, respectively. The function of *CYP2C19* in Psychiatry and its role in individualized medicine is also significant, especially with the long-term use of psychiatry medications. As we are in the era of precision medicine, the integration of *CYP2C19* genotyping into clinical decision-making is a crucial first step toward tailoring medications to specific genetic profiles of our patients, ultimately increasing the bar for patient care.
